# Effects of physiotherapeutic scoliosis-specific exercise in patients with mild juvenile scoliosis

**DOI:** 10.1186/s12891-022-05857-x

**Published:** 2022-10-15

**Authors:** Wangshu Yuan, Hai Wang, Keyi Yu, Jianxiong Shen, Lixia Chen, Ying Liu, Youxi Lin

**Affiliations:** 1 Department of Rehabilitation and Physical Therapy, Peking Union Medical College Hospital, Beijing, China; 2 Department of Orthopedic Surgery, Peking Union Medical College Hospital, Beijing, China

**Keywords:** Juvenile Idiopathic Scoliosis, Conservative Treatment, Physiotherapeutic Scoliosis Specific Exercise, The angle of trunk rotation

## Abstract

**Study design:**

A combined retrospective and prospective analysis on the therapeutic effect of physiotherapeutic scoliosis-specific exercise (PSSE) in mild juvenile idiopathic scoliosis (JIS) patients.

**Background:**

At present, patients with mild JIS are generally treated by observation without any interventional treatment. This study analyzed the effects of PSSE on mild JIS, which provided a new approach for the treatment of JIS.

**Method:**

A total of 52 patients with mild JIS (Cobb angle 10–19°), aged 4–9 years, self-selected into an observation group and a PSSE group. Patients performed the corrective posture exercises daily based on the Scientific Exercise Approach to Scoliosis (SEAS) to the best of their ability, and performed the over-corrective training based on Schroth methods for 30 min each day. Before and one year after the treatment, the Cobb angle and the angle of trunk rotation (ATR) were evaluated, and the results were compared between the two groups.

**Results:**

After one year of treatment, the Cobb angle in the PSSE group decreased from 15.0(11.0–17.0)° to 5.0(2.0–12.0)°(*p* ≤ 0.001), while the Cobb angle in the observation group increased from 13.5(11.0–17.3)° to 16.0(10.8–20.0)° (*p* = 0.010). The ATR in the PSSE group decreased from 5.0(2.0–7.0)° to 3.0(2.0–4.0)° (*p* = 0.009), while the change of ATR in the observation group was not significant. Compared with the observation group, 69.57% of patients in PSSE group had a decreased Cobb angle of more than 5 degrees, which was statistically significant(*p* ≤ 0.001).

**Conclusion:**

For mild JIS, PSSE decreased the Cobb angle and ATR.

**Supplementary Information:**

The online version contains supplementary material available at 10.1186/s12891-022-05857-x.

## Background

Idiopathic scoliosis is the most common spinal deformity in minors [1]. According to the age of onset, it is divided into infantile idiopathic scoliosis (IIS) (0–3 years), juvenile idiopathic scoliosis (JIS) (4–9 years) and adolescent idiopathic scoliosis (AIS) (10–18 years) [2]. Generally, clinical intervention was not suggested for idiopathic scoliosis during growth with Cobb angle < 20°, and observation with periodic follow-up was the main treatment method [3, 4]. However, with the improvement of people's health awareness and the development of physical therapy, patients with scoliosis and their parents are no longer satisfied with only observation, and physiotherapeutic scoliosis specific exercise (PSSE) is being increasingly used as an active treatment. PSSE is not a random training or sport, but involves specific exercises for patients with scoliosis according their unique curve pattern. There are several schools of PSSE in the Society on Scoliosis Orthopaedic and Rehabilitation Treatment (SOSORT), and the effectiveness of PSSE has been extensively evaluated [5]. Park [6] used meta-analysis to demonstrate that PSSE is an effective intervention for patients with scoliosis, and is more suitable for patients with Cobb angle < 30°. Monticone [7]  confirmed the validity of PSSE in a randomized controlled trial (RCT). However, the majority of the studies on PSSE were in AIS, while very few studies have been conducted on JIS [8, 9]. One possible reason for the focus of studies on AIS is that risk for curve progression is greater during adolescence. However, all JIS cases eventually undergo the whole period of rapid growth when they grow up. So, treating JIS at the earliest is critical. In this study, the efficacy of PSSE in JIS was evaluated.

## Materials and methods

### Subjects

All patients were enrolled at the Department of Physical Therapy and Rehabilitation between July 2017 and June 2018, and were diagnosed as JIS by orthopedic surgeons. Inclusion criteria were as follows: (1) 4–9 years old; (2) Cobb angle between 10–20°; (3) asymmetrical trunk and morphology compliance by X-ray. For example, patient with a left lumbar curve in the X-ray should also have a prominent appearance in the left lumbar; (4) females had not begun menstruation. The exclusion criteria were as follows: (1) received any intervention before the treatment, such as braces or PSSE; (2) history of spine surgery or injury; (3) patients with intellectual disability and those who were unable to understand the instructions; (4) Cobb angle < 20°, but parents strongly demanded brace treatment. A total of 52 patients met the study selection criteria and were recruited including 28 females and 24 males. The patients were self selected into the observation group (no intervention,28 patients) and the PSSE group (corrective position and over-corrective training,24 patients). The physical therapist (> 5 years experience in scoliosis conservative therapy) who conducted the exercise has obtained Schroth and SEAS certification. The PSSE group was asked to maintain the corrective position in their daily life, and performed the over-corrective exercise for > 30 min/day and > 5 days/week. All patients were asked to follow-up every 4–6 months. The patient was referred for a new X-ray if the ATR increased > 3° or trunk asymmetry increased during the follow-up. If the Cobb angle in the new X-ray was > 20° in the middle follow up, the patient was excluded from the study and recommended for brace treatment. In order to supervise the child's exercise, the physical therapist asked parents to upload photos of their child's training every two weeks. Before and after one year of treatment, the Cobb angle and the angle of trunk rotation (ATR) were evaluated.

### Evaluation and procedure

#### Cobb Angle [10]

The major Cobb angle was measured for the study. The Cobb angle was measured from the anterior–posterior X-rays by recording the angle between the upper and lower most-tilted end vertebra by a spine specialist/orthopedist (> 20 years experience in scoliosis operative treatment) and a physical therapist (PT) (> 5 years experience in scoliosis conservative therapy). The average of the two values was the eventual Cobb angle.

#### ATR [11]

The subjects were in standing position with 30 cm distance between the feet, and the PT sat behind them. The subjects were asked to bend forward slowly until the point when the deformity of the spine was most prominent and the top of the hump was parallel to the PT’s line of sight. The scoliometer (Mizuho OSI) was placed across the apical spinous process and the ATR angle was recorded. The major ATR was measured for the study.

### Treatment

The observation group did not receive any treatment and the patients were asked to follow-up every 4–6 months.

Using different SOSORT conservative treatment schools as references [12, 13], combined with our own experience, we administered conservative treatment based on the Peking Union Medical College (PUMC) classification [14]. In this study, PSSE consisted of two main aspects: corrective position and over-corrective training.

#### Corrective position

**Fig. 1:**
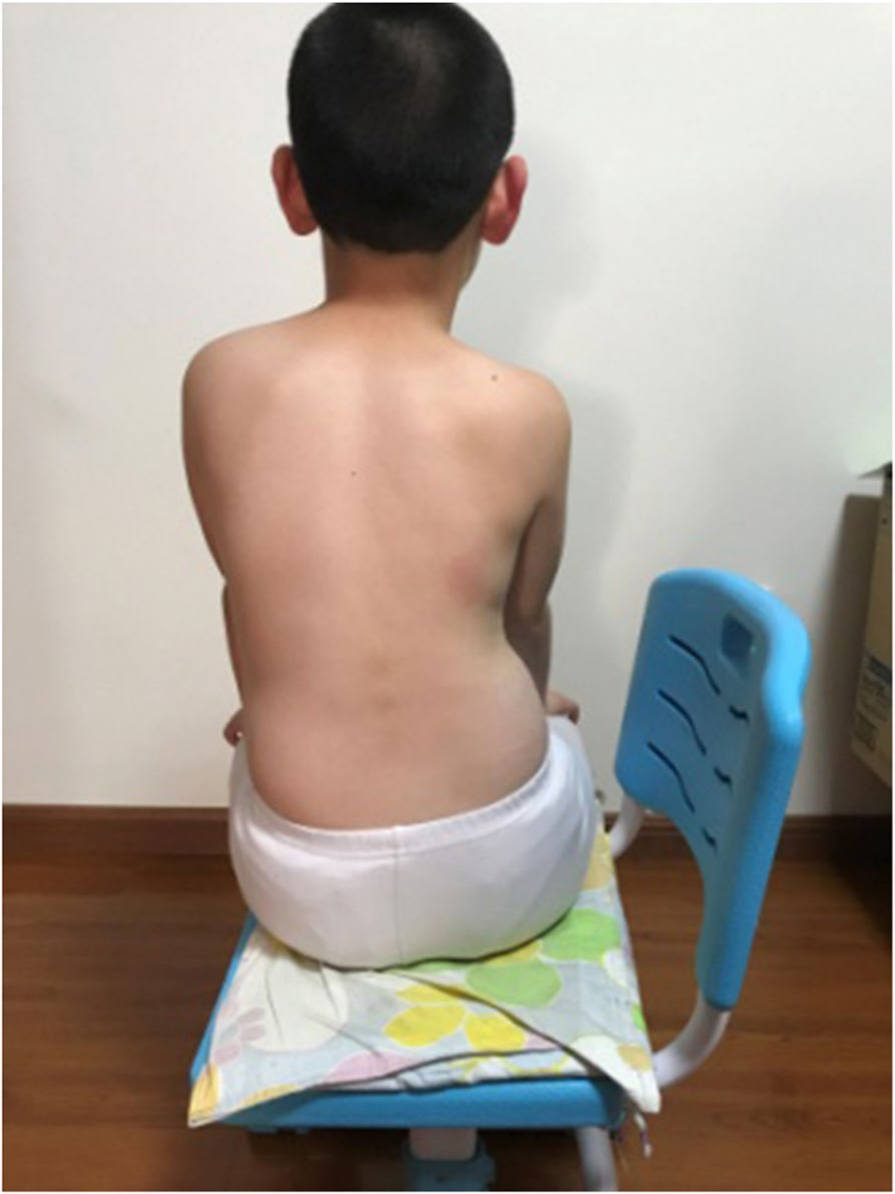
The patient in the habitual sitting posture

**Fig. 2:**
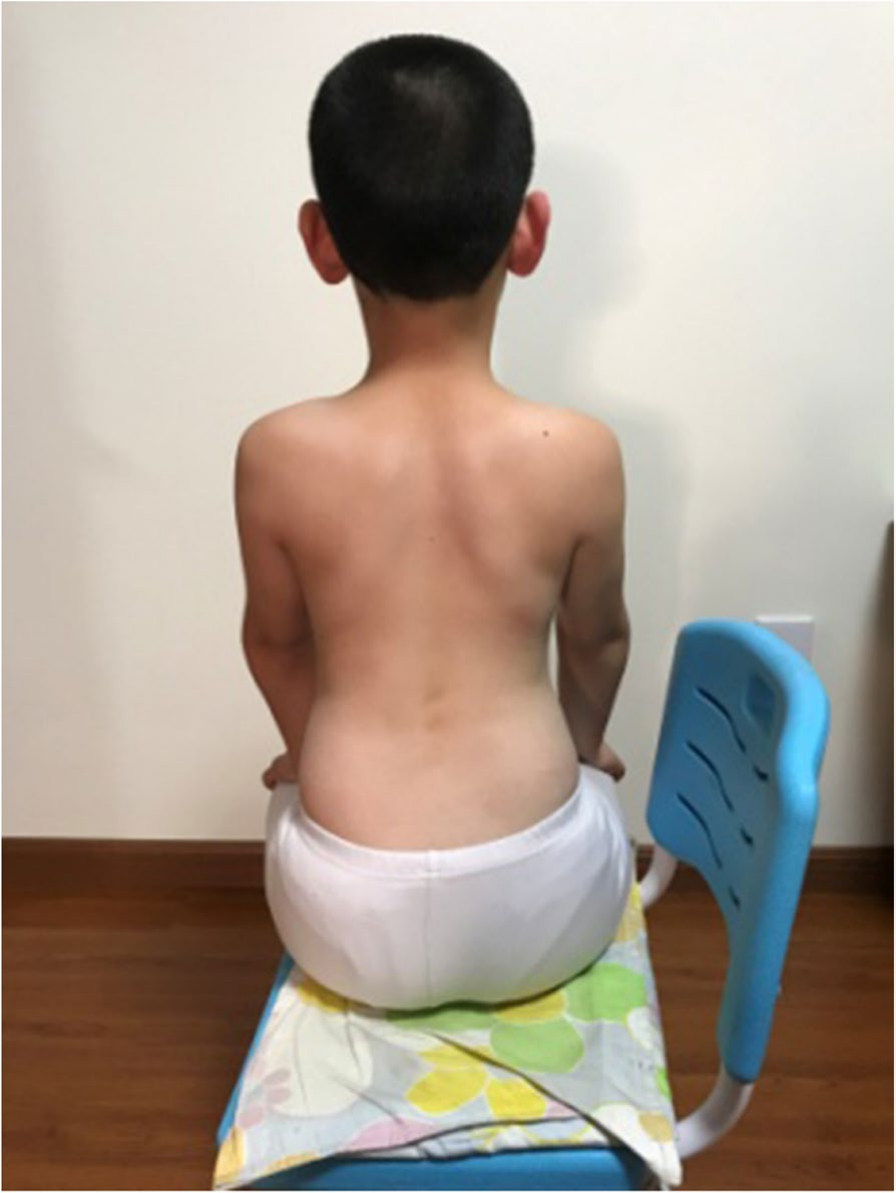
The patient in the corrective sitting posture

**Fig. 3:**
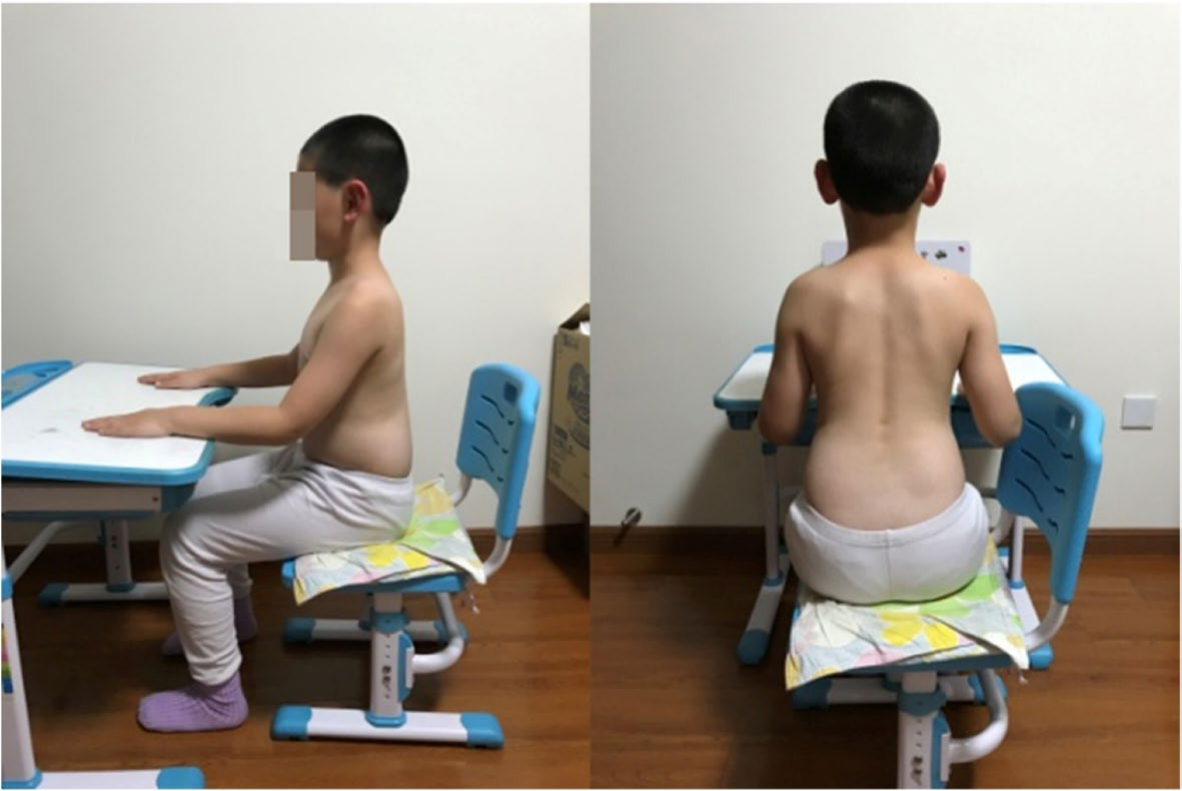
The patient pushed down on the table while maintain the corrective sitting position

The main concept of corrective position is derived from the SEAS approach. The PT taught the patients their specific three-dimensional corrective positions and how to ensure self-correction by themselves. The patient who had a left lumbar curve is shown in the habitual sitting posture in Fig. 1, and in the corrective sitting posture in Fig. 2. After the patient was in the corrective sitting position, the PT asked the patient to hold the corrective position and push down on the table or bend the torso forward/backward, in order to increase the stability of the corrective position (Fig. 3). Corrective sitting position is a daily training. Hence, the patients were expected to always maintain the corrective sitting position in daily life. If they could not control themselves actively all the time, they could use some help and do it in a passive way. For example, in this particular case, the patient could put a rice bag or a book under her/his convex side of the buttock. The patient with a left lumbar curve would place the pad on the left buttock. When the convex side of the buttock was boosted, the convex side of the pelvis was lifted, which passively pushed the curved spine to the neutral alignment. The corrective position also included standing and walking positions. In the standing position, the patient could lift her/his convex side of the pelvis or bend her/his concave side knee. In the walking position, the patient could put an insole in the shoe of her/his convex side. The aim was to lift the convex side pelvis to correct the spinal curve, and force the spine back to the neutral position. However, standing and walking corrective positions were not mandatory to complete in this study.

#### Over-corrective training

**Fig. 4:**
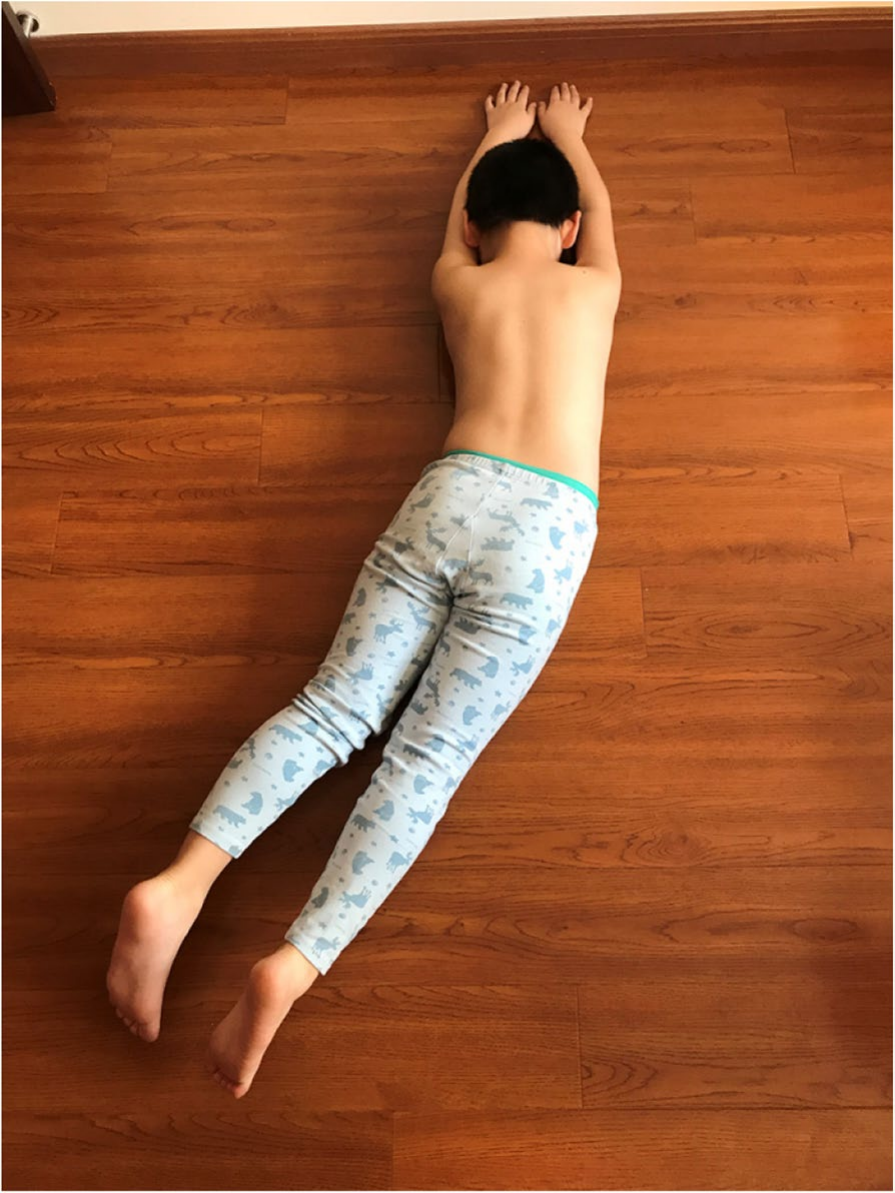
The patient in prone position, swung his lower limbs to the left and raised legs

**Fig. 5:**
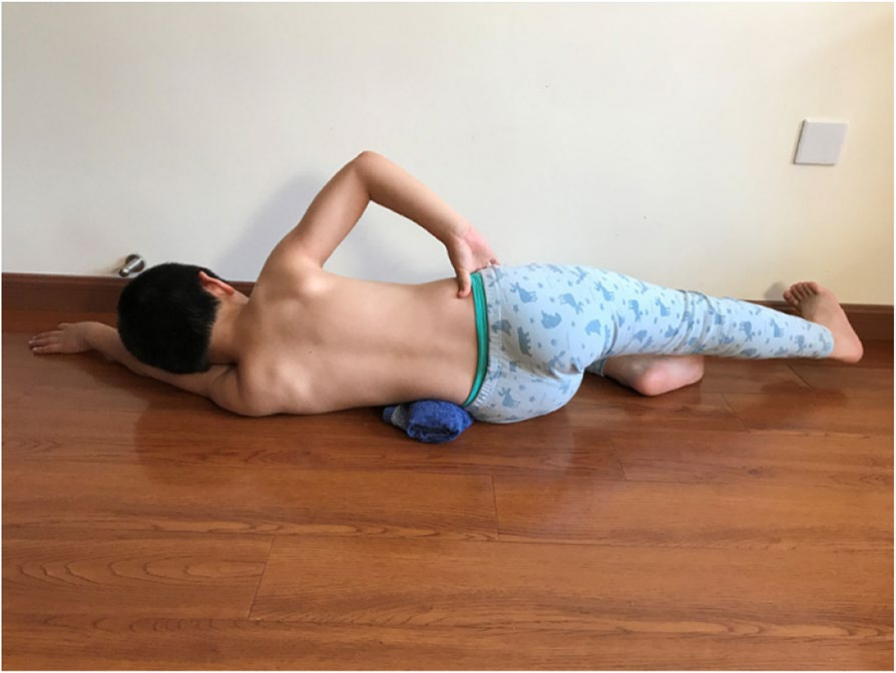
The patient lying on the left-side, lifted the right leg and stretched in caudal direction

**Fig. 6:**
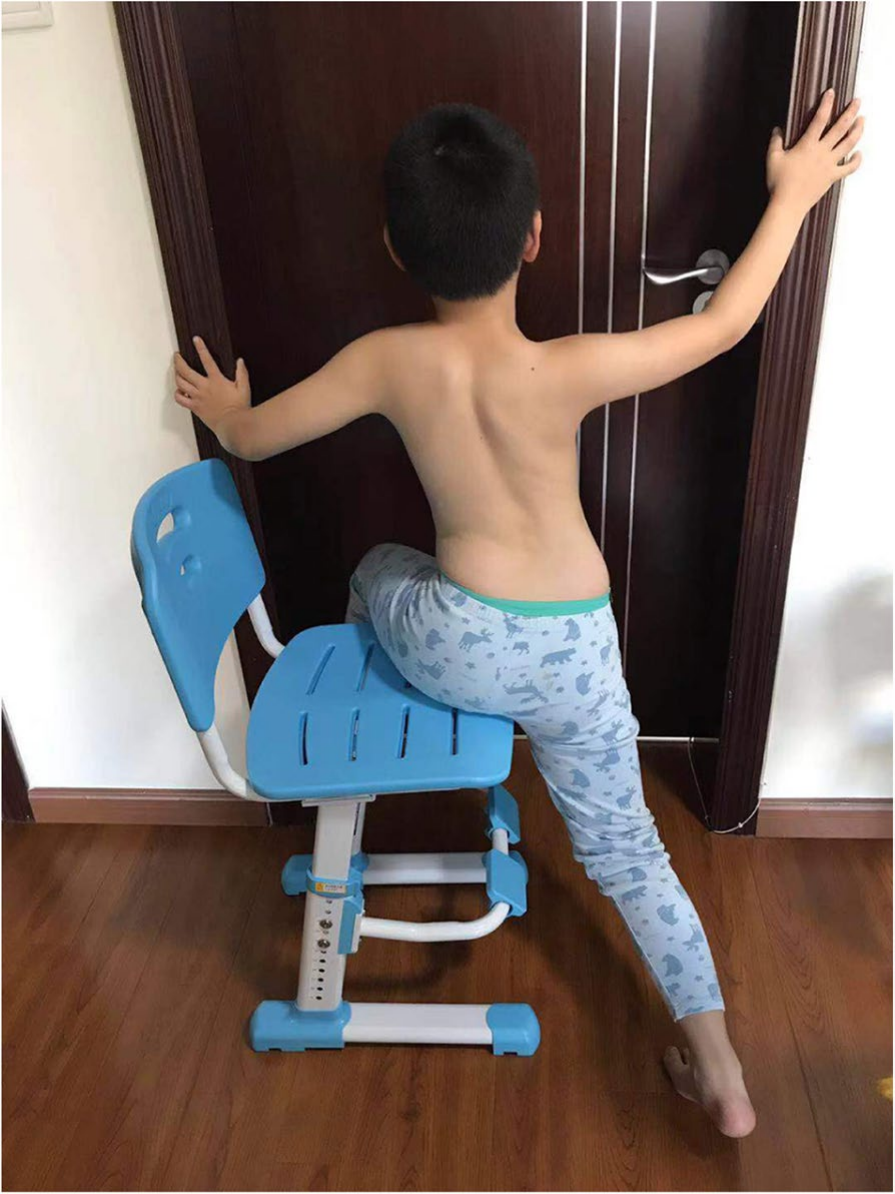
The patient pushed the chest to the upper-left and the right knee touched the ground

The main concept is derived from the Schroth approach. The JIS cases were classified according to the subtypes defined by the PUMC classification, Type I: Single Curve, Type II: Double Curve, Type III: Triple Curve. Different classifications had different over-corrective training schemes. During the over-corrective training, the patient's spine was forced to maintain the neutral position or even an over-corrective position. Taking the patient in Figure. 1 as an example, there were three main exercises included in the training: 1) the patient in prone position, swung his lower limbs to the left as much as he could and then raised the lower limbs as much as he could. Meanwhile, the upper body, upper limbs and head were kept still (Fig. 4). In this position, the lumbar would be pushed to the right, which reduced the Cobb angle. 2) the patient lay on the left side with a rice bag placed under his waist, and slightly lifted the right leg while ensuring that the right foot was lower than the hip and stretched the right leg in the caudal direction (Fig. 5). 3) the patient was in sitting position, with the left buttock on the chair and the right buttock hanging outside, the right thigh was perpendicular to the ground, the arms held the doorframe, the left shoulder attempted to touch the upper left corner of the door and the right knee was stretched to try to touch the ground (Fig. 6). The patients performed the training for 30 min/day, 5 days/week for one year.

### Statistical analysis

All statistical analyses were conducted using SPSS (version 19.0) software (SPSS, Chicago, IL). The measurement data were first analyzed using a normality test; those that obeyed the normal distribution were expressed as mean ± SD and analyzed using the paired-samples t test, whereas those data that did not obey the normal distribution were represented by the median (the first and third quartiles) and analyzed using either the Wilcoxon or the Mann–Whitney U test. The enumeration data were represented by the number of cases and the composition ratio, and analyzed using either the Pearson's chi-square test or Fisher's exact test. A *p*-value < 0.05 was considered statistically significant.

## Results

Before the treatment, there was no statistical difference in the age, gender, height, weight, body mass index, Cobb angle and ATR between the two groups (Table 1). Two patients in the observation group and one patient in the PSSE group were excluded because their Cobb angles were increased to > 20° in the 6 months follow-up visit and were referred for bracing.

**Table 1:** Descriptive analysis of the characteristics of the subjects in the two groups

Characteristic	Rang values	Observation Group	PSSE Group	*p*-value	t/z value
Sample size Age (years)	4–9	*n* = 26 7.0(6.0–9.0)	*n* = 23 8.0(7.0–9.0)	0.297	-1.044
Height (cm)	109.5–156.5	131.5(123.5–140.0)	135.0(126.0–138.5)	0.616	-.501
Weight (kg)	18.5–54	27.0(22.9–33.0)	27.0(24.5–29.5)	0.802	-.251
BMI (Kg/m^2^)	11.5–23.9	15.8 (14.6–18.4)	14.5(14.1–15.8)	0.052	-1.943
Cobb angle(degree)	10–19	13.5(11.0–17.3)	15.0(11.0–17.0)	0.672	-.423
ATR(degree)	0–9	4.0 ± 2.5	4.4 ± 2.7	0.633	-.481

### The Cobb angle before and after treatment

After one year of treatment, the Cobb angle in the PSSE group significantly decreased(*p* ≤ 0.001), while the Cobb angle in the observation group significantly increased (*p* = 0.010) (Table 2).

**Table 2:** Results of the analysis of the Cobb angle in the two groups$\left({\overline x\pm s}^\circ\right)$

Group	Before the treatment	After the treatment	*p*-value	z value
Observation	13.5(11.0-17.3)	16.0(10.8-20.0)	0.010^*^	- 2.564
PSSE	15.0(11.0-17.0)	5.0(2.0-12.0)	≤0.001^*^	- 3.646
p-value			≤0.001^○^	
t value				-3.847

^*^Differences within the same group were significant, *p* < 0.05

^○^Difference between the two groups after the treatment was significant, *p* < 0.05

In PSSE group, the Cobb angle increased by > 5° in one patient and decreased by > 5° in 16 patients. In observation group, the Cobb angle increased by > 5° in 9 patients, and no patient's Cobb angle decreased by > 5° (Table 3).

**Table 3:** Analysis of patients in each group whose Cobb angles increased or decreased beyond the threshold of 5 degrees

	Cobb angle increased by > 5°	Cobb angle decreased by > 5°
Observation group (Age and the variation value of Cobb angle)	The number of patients is nine (34.62% of 26 patients.)	The number of patients is zero
	9 years old, 19° to 25° 9 years old, 19° to 25° 9 years old, 18° to 25° 8 years old, 18° to 24° 9 years old, 11° to 17° 7 years old, 11° to 18° 9 years old, 10° to 17° 9 years old, 11° to 17° 9 years old, 10° to 16°	
PSSE group (Age and the variation Value of Cobb angle)	The number of patients is one	The number of patients is sixteen (69.57% of 23 patients)
	9 years old, 19° to 26°	7 years old, 16° to 0° 9 years old, 11° to 0° 9 years old, 16° to 5° 9 years old, 15° to 9° 8 years old, 17° to 3° 8 years old, 11° to 0° 9 years old, 15° to 4° 6 years old, 10° to 0° 9 years old, 10° to 0° 9 years old, 15° to 2° 9 years old, 10° to 2° 6 years old, 10° to 4° 8 years old, 16° to 10° 9 years old, 19° to 12° 7 years old, 18° to 10° 9 years old, 15° to 5°

Compared with the observation group, 69.57% of patients in PSSE group had a decreased Cobb angle of more than 5 degrees, which was statistically significant(*p* ≤ 0.001) (Table 4).

**Table 4:** Results of the analysis ofpatients whose Cobb angle decreased by > 5° in the two groups

Group	The number of Cobb angle decreased by > 5°(%)	The number of Cobb angle decreased by ≤ 5°(%)	Total
Observation	0(0)	26(100)	26
PSSE	16(69.57)	7(30.43)	23
Total	16	33	49
*p*-value			≤ 0.001^*^
χ^2^ value			26.856

^*^Difference between the two groups after the treatment was significant, *p* < 0.05

### The ATR before and after treatment

After one year of treatment, the ATR in the PSSE group significantly decreased (*p* = 0.009) (Table 5).

**Table 5:** Results of the analysis of the ATR in the two groups ($\overline{x } \pm s$°)

Group	Before the treatment	After the treatment	*p*-value	t/z value
Observation	4.0±2.5	4.8±3.0/5.0(2.8-7.3)	0.067	-1.912
PSSE	5.0(2.0-7.0)	3.0(2.0-4.0)	0.009^*^	-2.611
p-value			0.042^○^	
z value				-2.035

^*^Differences within the same group were significant, *p* < 0.05

^○^Difference between the two groups after the treatment was significant, *p* < 0.05

## Discussion

Compared with IIS and AIS, JIS has received the least attention in clinical field. There are many studies on the natural history of idiopathic scoliosis [15–17], however few of them considered JIS alone. Fusco C [19] conducted a retrospective cohort observational study in JIS and found that conservative treatment initiated already in childhood may favorably change the natural history of JIS with the aim of reaching a curve as far as possible from surgical thresholds. Di Felice F [18]  performed a meta-analysis and found high progression rates of 49% (95% CI = 1–97%) in IIS, 49% (95% CI = 19–79%) in a mixed group of patients with JIS or AIS, and 42% (95% CI = 11–73%) in AIS. Our study found that the Cobb angle of nine JIS patients (34.62%) in observation group increased by > 5° within one year, and their common characteristics were older age(close to 9 years old). It suggested that we should focus on JIS patients who are about to enter a rapid growth period and review them regularly. In the observation group, one third of patients' Cobb angle increase by > 5° and no patients' Cobb angle decrease by > 5° after one year's observation. This is an indication that in the natural process of JIS, few patient's Cobb angle decrease naturally, and 30% cases have the risk of aggravation. In this study, patients who received PSSE treatment achieved an improved Cobb angle not only in statistically significant but also in clinically. It proved that PSSE could be used as an effective treatment to reduce the Cobb angle of mild JIS. The increase of Cobb angle in observation group was statistically significant rather than clinically significant. It shows that JIS is progressive at a slow but real pace. Clinically significant may be found if the observation period is prolonged. Compared with the observation group, patients who received PSSE treatment achieved an improved ATR in statistically significant but not in clinically after one year treatment. According to the instruction manual of scoliometer, a change of 3 degree or more of a scoliometer measurement indicates possible curve progression. A change of 2 degree or less usually indicates only minor variation in posture. Our analysis is that ATR mainly reflects the rotation of vertebral body and the deformity of ribs. These two problems are not serious in mild JIS patients, so there is no obvious clinical significance. Overall, our findings suggest that PSSE may control or even reduce the progression of the disease. Hence, it may be important to reconsider if observation is an appropriate treatment for mild JIS.

SOSORT has reached a consensus that PSSE is an effective treatment for patients, and SOSORT experts have agreed that PSSE should consist of three-dimensional auto-correction, training in activities of daily living (ADL), stabilizing the corrected posture and patient education [19]. In this study, PSSE treatment included two important aspects: corrective position and over-corrective training. The corrective position is suitable for the daily life. The etiology of JIS is unclear, but in JIS, the curve will cause an uneven load on the vertebra and vice-versa [20]. The majority of the patients with JIS are students, who sit all day, and an incorrect position, especially during sitting, will increase the Cobb angle. The corrective position is an effective method to realign the spine and break this vicious cycle [21]. Given that patients with JIS are too young to always perform the self-correction by themselves, using a rice bag as a passive way to force the spine back to the neutral alignment is an effective treatment. Combined with the over-corrective training, which is focused on the over-correction and muscle strength exercise, the Cobb angle and ATR of the patients improved in this study. Most of the studies on PSSE in idiopathic scoliosis were conducted in adolescents, because patients with AIS have the highest progressive risk during puberty. Monticone [7] found that PSSE could reduce spinal deformities and enhance the health-related quality of life in patients with mild AIS. The effects lasted for at least one year after the intervention. Zapata [22]enrolled 49 patients with AIS to study the effects of PSSE for one year. The results showed that the PSSE group had lesser curve progression as compared to the controls. Negrini [23] conducted a longitudinal comparative observational multi-center study, which included 327 consecutive patients, and found that PSSE reduced the bracing rate in AIS and was more effective than standard physiotherapy. PSSEs are additional tools that can be included in the therapeutic regimen for AIS. Using these studies as a reference, we considered that compliance is the most important factor for the effectiveness of PSSE. There are many different PSSEs, and there is no consensus on the best one. Although there are very few studies on JIS, we need to actively treat JIS because all JIS cases will undergo the period of rapid growth eventually, and then suffer the high risk of progression. In this study, when patients complete the PSSE in accordance with the requirements, 16(69.57%) of them achieved a positive result(Cobb angle decreased more than 5 degree). If the spine deformity in JIS can be reduced, it will provide a better basis for AIS in the future.

## Conclusion and limitations

The results indicated that the PSSE was effective in reducing the Cobb angle and ATR in juveniles with mild idiopathic scoliosis. Thus, corrective position and over-corrective training should be helpful in treating patients with JIS.

However, this study also has some obvious limitations. First, it's not a RCT and participants chose their groups according to their wishes. In order to make the research more rigorous, we plan to conduct randomized controlled studies in the future. Second, SRS-22 questionnaire is a health related quality of life(HRQOL) tool for AIS [24]. Although we have done some research on JIS' HRQOL according to SRS-22 in this study, we couldn't use SRS-22 directly. A HRQOL questionnaire should be developed for JIS in the future. Third, for patients with significantly reduced Cobb angle in PSSE group, we couldn't summarize their common characteristics. What kind of JIS patients are more sensitive to PSSE is the next step to be studied. Last, this study has only followed patients for one year which is still too short for JIS, and we will conduct a study with a longer observation in the future.

## Supplementary Information

**Additional file 1: Supplementary table 1.** Data of the subjects in the observation group

**Additional file 2: Supplementary table 2.** Data of the subjects in the PSSE group

## Data Availability

The datasets used and/or analyzed in the current study are available from the corresponding author upon reasonable request.

## Abbreviations

SEAS: Scientific Exercise Approach to Scoliosis
PSSE: Physiotherapeutic scoliosis-specific exercise
JIS: Juvenile idiopathic scoliosis
SRS-22: Scoliosis Research Society Patient Questionnaire-22
ATR: Angle of trunk rotation
AIS: Adolescent idiopathic scoliosis
SOSORT: Society on Scoliosis Orthopaedic and Rehabilitation Treatment
RCT: Randomized controlled trial
IIS: Infantile idiopathic scoliosis
PT: Physical therapist
PUMC: Peking Union Medical College
BMI: Body mass index
ADL: Activities of daily living
HRQOL: Health related quality of life
