# Supplementary material for: Effects of physiotherapeutic scoliosis-specific exercise in patients with mild juvenile scoliosis
Source: BMC Musculoskelet Disord. 2022 Oct 15;23:918. doi: 10.1186/s12891-022-05857-x (PMC9569063; doi:10.1186/s12891-022-05857-x)
Supplement: Supplementary file 1 — Additional file 1: Supplementary table 1. Data of the subjects in the observation group [file 12891_2022_5857_MOESM1_ESM.doc]

Supplementary table 1. data of the subjects in the observation group

| No. | age | gender, male=1,female=2 | weight | height | BMI | Cobb angle before treatment | Cobb angle after one year treatment | ATR before treatment | ATR after one year treatment |
| --- | --- | --- | --- | --- | --- | --- | --- | --- | --- |
| 1 | 9 | 2 | 27 | 139.5 | 13.87444 | 19 | 25 | 6 | 9 |
| 2 | 9 | 1 | 54 | 156.5 | 22.04779 | 19 | 25 | 5 | 9 |
| 3 | 9 | 2 | 27 | 136.5 | 14.491 | 18 | 25 | 8 | 10 |
| 4 | 8 | 2 | 23 | 131 | 13.40248 | 18 | 24 | 7 | 8 |
| 5 | 9 | 2 | 31 | 143 | 15.15967 | 11 | 17 | 4 | 6 |
| 6 | 7 | 2 | 29 | 135 | 15.91221 | 11 | 18 | 4 | 6 |
| 7 | 9 | 1 | 40 | 141.5 | 19.97777 | 10 | 17 | 2 | 4 |
| 8 | 9 | 2 | 33 | 142 | 16.3658 | 11 | 17 | 2 | 5 |
| 9 | 9 | 2 | 51 | 151 | 22.36744 | 10 | 16 | 3 | 6 |
| 10 | 9 | 1 | 33 | 137.5 | 17.45455 | 13 | 9 | 2 | 2 |
| 11 | 6 | 1 | 27 | 125 | 17.28 | 12 | 8 | 4 | 0 |
| 12 | 6 | 1 | 21 | 120 | 14.58333 | 18 | 23 | 7 | 8 |
| 13 | 6 | 1 | 33 | 123.5 | 21.63615 | 15 | 10 | 2 | 0 |
| 14 | 7 | 2 | 24 | 127 | 14.88003 | 16 | 15 | 8 | 7 |
| 15 | 8 | 2 | 26 | 134 | 14.47984 | 19 | 24 | 6 | 7 |
| 16 | 7 | 1 | 26 | 127.5 | 15.99385 | 16 | 11 | 6 | 3 |
| 17 | 8 | 2 | 37 | 141.5 | 18.47944 | 13 | 10 | 2 | 1 |
| 18 | 4 | 2 | 22.5 | 110 | 18.59504 | 14 | 16 | 4 | 5 |
| 19 | 7 | 1 | 26 | 131.5 | 15.03564 | 10 | 15 | 2 | 4 |
| 20 | 9 | 1 | 30 | 132 | 17.21763 | 12 | 10 | 0 | 0 |
| 21 | 6 | 1 | 21 | 124 | 13.65765 | 17 | 13 | 8 | 5 |
| 22 | 6 | 1 | 27 | 131.5 | 15.61393 | 11 | 16 | 3 | 4 |
| 23 | 4 | 1 | 22 | 109.5 | 18.34824 | 14 | 18 | 3 | 5 |
| 24 | 5 | 1 | 18.5 | 116 | 13.74851 | 13 | 11 | 0 | 4 |
| 25 | 7 | 2 | 24 | 123.5 | 15.73538 | 12 | 8 | 1 | 0 |
| 26 | 5 | 2 | 18.8 | 110 | 15.53719 | 15 | 19 | 6 | 8 |
