# Supplementary material for: Effects of physiotherapeutic scoliosis-specific exercise in patients with mild juvenile scoliosis
Source: BMC Musculoskelet Disord. 2022 Oct 15;23:918. doi: 10.1186/s12891-022-05857-x (PMC9569063; doi:10.1186/s12891-022-05857-x)
Supplement: Supplementary file 2 — Additional file 2: Supplementary table 2. Data of the subjects in the PSSE group [file 12891_2022_5857_MOESM2_ESM.docx]

Supplementary table 2. data of the subjects in the PSSE group

| No. | age | gender, male=1,female=2 | weight | height | BMI | Cobb angle before treatment | Cobb angle after one year | ATR before treatment | ATR after one year treatment |
| --- | --- | --- | --- | --- | --- | --- | --- | --- | --- |
| 1 | 9 | 1 | 32 | 138.5 | 16.68209 | 19 | 26 | 9 | 11 |
| 2 | 7 | 2 | 19 | 128.5 | 11.50661 | 16 | 0 | 5 | 2 |
| 3 | 9 | 2 | 29.5 | 135 | 16.18656 | 11 | 0 | 3 | 2 |
| 4 | 9 | 2 | 27.5 | 141 | 13.8323 | 16 | 5 | 5 | 4 |
| 5 | 9 | 1 | 38 | 126 | 23.9355 | 15 | 9 | 5 | 3 |
| 6 | 8 | 1 | 40 | 134.5 | 22.11136 | 17 | 3 | 7 | 4 |
| 7 | 8 | 1 | 24.5 | 131 | 14.27656 | 11 | 0 | 4 | 3 |
| 8 | 9 | 2 | 25 | 136.5 | 13.4176 | 15 | 4 | 7 | 3 |
| 9 | 6 | 2 | 24.5 | 120 | 17.01389 | 10 | 0 | 3 | 2 |
| 10 | 9 | 2 | 29 | 135.5 | 15.79499 | 15 | 2 | 6 | 3 |
| 11 | 9 | 1 | 32.5 | 144 | 15.67323 | 10 | 2 | 4 | 2 |
| 12 | 6 | 1 | 19.5 | 117 | 14.24501 | 10 | 4 | 0 | 0 |
| 13 | 8 | 1 | 31 | 141 | 15.59278 | 16 | 10 | 2 | 1 |
| 14 | 9 | 1 | 27 | 138 | 14.17769 | 19 | 12 | 9 | 4 |
| 15 | 7 | 2 | 26 | 131.5 | 15.03564 | 18 | 10 | 5 | 3 |
| 16 | 9 | 2 | 27 | 139.5 | 13.87444 | 15 | 5 | 0 | 0 |
| 17 | 9 | 2 | 25 | 135 | 13.71742 | 10 | 0 | 2 | 1 |
| 18 | 7 | 2 | 28 | 133.5 | 15.7107 | 16 | 15 | 5 | 6 |
| 19 | 5 | 2 | 21 | 120.5 | 14.46256 | 19 | 19 | 7 | 8 |
| 20 | 7 | 1 | 29 | 138 | 15.22789 | 14 | 10 | 1 | 1 |
| 21 | 9 | 1 | 28 | 140 | 14.28571 | 13 | 10 | 2 | 2 |
| 22 | 4 | 1 | 19 | 115 | 14.36673 | 12 | 12 | 2 | 2 |
| 23 | 5 | 2 | 20 | 119 | 14.1233 | 17 | 17 | 8 | 9 |
